# Structural biases in digital health intervention and health equity research: a bibliometric knowledge graph analysis (2015–2025)

**DOI:** 10.3389/fdgth.2026.1883026

**Published:** 2026-09-01

**Authors:** Shengtao Li, Zhongyue Li, Cheng Chen, Wu Chen, Wenqian Du, Xiaolin Yao

**Affiliations:** 1 Graduate School, Harbin Sport University, Harbin, China; 2 Clinical Medical College, Hubei Enshi University, Enshi, China; 3 School of Sports Humanities and Society, Harbin Sport University, Harbin, China

**Keywords:** digital health intervention, health equity, mHealth, telemedicine, wearable devices

## Abstract

**Objective:**

This study systematically analyzed the knowledge structure, evolution patterns, and structural biases in the intersection of digital health interventions, patient engagement/adherence, and health equity from 2015 to 2025.

**Methods:**

We searched Scopus, Web of Science Core Collection, and PubMed. We included 4,677 original articles or systematic reviews. We used Bibliometrix, SciExplorer, and CiteSpace for bibliometric and knowledge graph analysis.

**Results:**

The annual number of publications increased from 75 to 1,317 (R^2^ = 0.9955). The average annual growth rate was 33.18%. However, author keywords were highly dispersed (9,302 vs. 4,971). Geographical distribution showed “one superpower and multiple strong countries.” The United States accounted for more than 54% of the top ten countries’ frequencies. China ranked fifth but did not enter the core collaboration network. JMIR series journals published 58.8% of the top ten journals’ total articles. Keyword clustering identified ten themes, including telemedicine and chronic disease management. “Health equity” did not become an independent high-frequency cluster. The author collaboration network showed a multi-center structure with methodologists, theory builders, and tool developers as cores.

**Conclusion:**

This field faces “knowledge disorder under exponential growth.” There are systematic biases in geography, theme, and power structure. We recommend building a core literature database, creating interdisciplinary journals, supporting reverse innovation led by low- and middle-income countries, and reforming academic evaluation systems to bridge the gap between research and policy translation.

## 1 Introduction

The rapid spread of digital health interventions is changing how healthcare is delivered and how patients manage their health. Technologies like mobile health apps, wearable devices, and remote monitoring systems are expected to improve access to care, optimize chronic disease management, and reduce costs (1, 2). However, introducing technology does not automatically improve health equity. If interventions are not designed for vulnerable groups, digital health may worsen existing health inequalities and amplify the “digital divide” (3, 91). The World Health Organization clearly stated in its Global Digital Health Strategy (2020–2025) that one core goal of digital health is “to promote universal health coverage and equity” (4). Yet, how to empirically evaluate the combined impact of digital health interventions on patient engagement, treatment adherence, and health equity remains a frontier challenge (2).

Previous studies have accumulated rich evidence in many areas. For example, mobile health interventions have shown moderate positive effects on medication adherence for hypertension, diabetes, and HIV/AIDS (5). Telemedicine applications in mental health services have been shown to reduce geographic barriers and improve access (6, 96). At the same time, more studies are focusing on equity issues in digital health. They find that people with low education, low income, older adults, rural residents, and ethnic minorities face significant disadvantages in accessing and using digital health technologies (7, 8, 97). Some studies point out that without intervention, digital health may become an area where “the rich benefit and the poor are excluded,” violating the fundamental ethics of public health (9, 98).

Recent umbrella reviews have synthesized evidence on digital health interventions and health equity, identifying causal pathways and mapping existing systematic reviews onto an intervention logic model (1, 10). Bitomsky et al. (11) further systematically reviewed the role of digital health technologies in advancing health equity.

Despite these contributions, the existing evidence system has obvious fragmentation. First, most studies focus on a single disease or a single technology platform. They lack an overall perspective on the systematic connections among digital health interventions, patient engagement behaviors, and health equity. Second, most study designs are randomized controlled trials and cross-sectional surveys. Few use bibliometric methods that can reveal the evolution of knowledge structures. Third, although several systematic reviews have discussed digital health effectiveness or equity issues, no study has systematically mapped the field's time series, geographic distribution, interdisciplinary patterns, collaboration networks, and research hotspot evolution from a macro level. This “cannot see the forest for the trees” limitation makes it hard for policymakers, funders, and researchers to understand the full picture, frontiers, and blind spots of the field (12).

Bibliometrics provides powerful tools to address these problems. By quantitative and visual analysis of large-scale literature data (13), researchers can reveal a discipline's knowledge base, evolution path, core journals, productive countries, collaboration networks, and research frontiers. In recent years, tools like Bibliometrix, SciExplorer, and CiteSpace have been widely used in public health, nursing, and medical informatics (12, 14), but they are rarely applied to the intersection of digital health interventions and health equity. Based on this, this study takes “digital health intervention,” “patient engagement/adherence,” and “health equity” as core concepts. We systematically searched Scopus, Web of Science Core Collection, and PubMed for original research and systematic reviews published from 2015 to 2025. We used bibliometric and knowledge graph analysis to: (1) describe annual publication trends, geographic distribution, and core journals in this field; (2) identify clusters of research hotspots and thematic evolution paths; (3) reveal interdisciplinary patterns, author collaboration networks, and knowledge power structures; and (4) reflect on the gap between academic prosperity and policy translation based on the above analyses, then propose future research priorities and methodological suggestions. The results will provide important references for evidence synthesis, research design, and policy interventions in the field of digital health equity.

Recent bibliometric studies have mapped the evolution of global digital health research, identifying four major thematic fronts—systems-strengthening in LMICs, digital epidemiology and algorithmic equity, digital-health literacy and evidence-based eHealth, and virtual-care transformation during COVID-19—while highlighting cross-cutting gaps in interoperability, sustainability, and LMIC-led evaluation (Megatrends review, 2026). Similarly, hybrid scoping and bibliometric reviews have examined the effectiveness, equity, and implementation challenges of machine learning in population health management (2026) (15).

Based on this, this study takes “digital health intervention,” “patient engagement/adherence,” and “health equity” as core concepts. We systematically searched Scopus, Web of Science Core Collection, and PubMed for original research and systematic reviews published from 2015 to 2025. Using bibliometric and knowledge graph analysis, this study aims to answer the following four research questions:

**RQ1 (Descriptive)**: What are the annual publication trends, geographic distribution, and core journal characteristics in this field from 2015 to 2025?
**RQ2 (Structural)**: What are the clusters of research hotspots and the paths of thematic evolution?
**RQ3 (Relational)**: What are the interdisciplinary patterns, author collaboration networks, and knowledge power structures?
**RQ4 (Reflective)**: Based on the above analyses, what is the gap between academic prosperity and policy translation? What are the future research priorities and methodological recommendations?

The results will provide important references for evidence synthesis, research design, and policy interventions in the field of digital health equity.

## 2 Materials and methods

This study searched three databases (Scopus, Web of Science Core Collection, and PubMed) for original research or systematic reviews published from January 1, 2015 to December 31, 2025. We chose this time range for several reasons. Around 2015, mobile health (mHealth), wearable devices, and digital intervention technologies entered a rapid development phase (16–18). At the same time, health equity and digital divide issues received wide attention in public health. The last decade of literature can well reflect the progress and evolution of this field (19, 20).

### 2.1 Databases and search strategy

The detailed search strategy is shown in Table 1. The search strategy was built around three core concept groups: digital health intervention, patient engagement/adherence, and health equity. We used the Boolean operator OR to combine keywords within each group to broadly cover related terms. We used AND to connect the three groups, so that each retrieved document covered all three core domains, meeting the integration needs of this study.

**Table 1:** Literature search strategy.

Search Category	Keywords	Boolean Operators	Search Scope	Reason for Selection
Digital health intervention	digital health, mobile health, mHealth, eHealth, wearable, wearables, smartphone, mobile application, app, digital intervention, telemedicine, remote monitoring	OR	Title, Abstract, Keywords (Scopus: TITLE-ABS-KEY; PubMed: Title/Abstract; WoS: Topic)	Covers core technology forms of digital health interventions (mobile health, wearables, smartphone apps, remote monitoring, etc.), ensuring comprehensive capture of exposure variables
Patient engagement/adherence	patient adherence, patient engagement, treatment adherence, medication adherence, health behavior, self-management, participation, retention, dropout, attrition, engagement"	OR	Same as above	Covers behavioral indicators such as patient adherence, engagement, self-management, retention and attrition, accurately reflecting participation and outcome variables during intervention
Health equity	health equity, digital divide, digital health divide, digital inequality, health disparity, health inequality, social determinant, vulnerable population, underserved, low income, low education, rural, minority, access to care, fairness, equitable, PROGRESS, disparity, socioeconomic, education level, digital literacy	OR	Same as above	Covers health equity and digital divide-related social determinants (income, education, geography, ethnicity, etc.), ensuring inclusion of literature on vulnerable populations and equity analysis
Combined search strategy	(Digital health intervention terms) OR (Patient engagement/adherence terms) OR (Health equity terms)	OR	Same as above	Ensures that retrieved literature simultaneously addresses all three core domains (digital intervention, patient engagement/adherence, health equity)

Specifically, the “digital health intervention” search terms included: digital health, mobile health, mHealth, eHealth, wearable, wearables, smartphone, mobile application, app, digital intervention, telemedicine, remote monitoring. The “patient engagement/adherence” terms included: patient adherence, patient engagement, treatment adherence, medication adherence, health behavior, self-management, participation, retention, dropout, attrition, engagement. The “health equity” terms included: health equity, digital divide, digital health divide, digital inequality, health disparity, health inequality, social determinant, vulnerable population, underserved, low income, low education, rural, minority, access to care, fairness, equitable, PROGRESS, disparity, socioeconomic, education level, digital literacy.

Field limits were as follows: Scopus used TITLE-ABS-KEY; Web of Science used Topic (TS); PubMed used Title/Abstract. We chose three databases for complementary subject coverage, bibliometric needs, and balance between recall and efficiency. PubMed focuses on core biomedical and public health literature. Web of Science adds social sciences and interdisciplinary studies (such as health behavior interventions). Scopus further expands engineering, technology, and health services research. All three provide standardized citation data to support subsequent collaboration network and keyword clustering analyses. Together, the three databases cover more than 85% of peer-reviewed journals in this field (21), consistent with systematic review methodology conventions. The final retrieval results were: Scopus 6,042, Web of Science 4,335, PubMed 644.

### 2.2 Data screening

Inclusion and Exclusion Criteria. We applied the following inclusion criteria: (a) original research articles or systematic reviews; (b) published between January 1, 2015 and December 31, 2025; (c) written in English; (d) indexed in Scopus, Web of Science Core Collection, or PubMed; and (e) containing at least one keyword from each of the three core concept groups (digital health intervention, patient engagement/adherence, and health equity) in the title, abstract, or keywords (22).

Exclusion criteria were: (a) editorials, commentaries, letters, conference abstracts, book chapters, and dissertations; (b) non-English publications; (c) duplicate records across databases; and (d) papers that mentioned digital health or health equity only superficially (e.g., in the discussion as a future direction without empirical analysis) (23).

**Figure 1:**
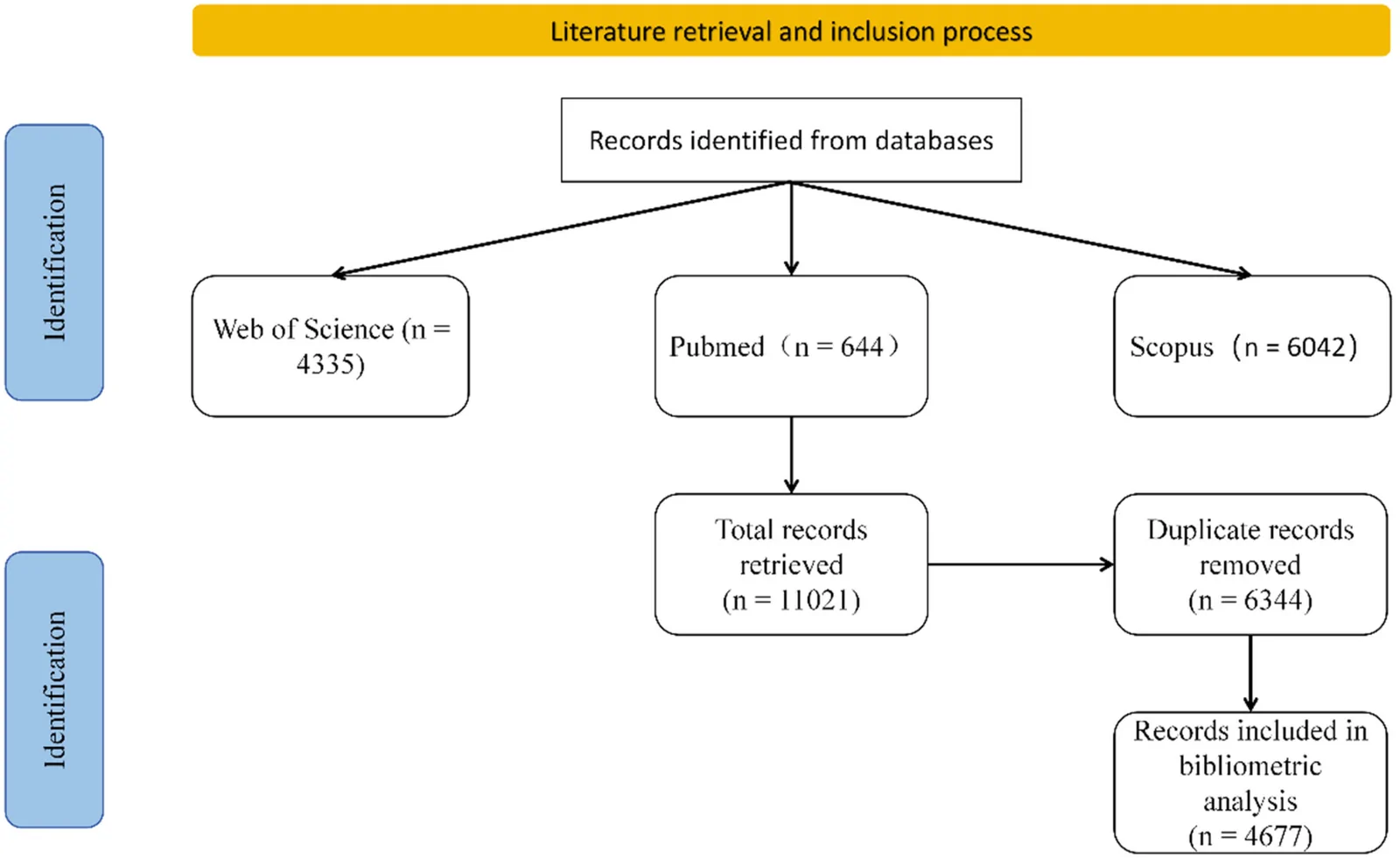
Literature retrieval and screening flowchart.

Figure 1 presents the literature screening flowchart. The search was performed in **March 2025**. In the retrieval phase, we executed the above search strategy in Scopus, Web of Science Core Collection, and PubMed. The time range was 2015–2025. Document types were limited to original articles or systematic reviews. The language was limited to English. The raw results were: Scopus 6,042, Web of Science 4,335, PubMed 644, totaling 11,021. In the deduplication phase, we merged the results into reference management software. We used titles, authors, journals, publication years, and DOIs to identify and remove duplicate records. After deduplication, we removed 6,344 duplicates and obtained 4,677 documents for subsequent bibliometric analysis and systematic review.

### 2.3 Data analysis methods

#### 2.3.1 Rationale for choosing bibliometric analysis

The choice of bibliometric analysis as the primary knowledge synthesis method in this study is justified by three considerations. First, the field has accumulated 4,677 documents over the past decade, with an annual growth rate of 33.18%—far exceeding the capacity of traditional narrative reviews or manual close reading. Bibliometric methods enable researchers to systematically reveal a discipline's knowledge base, evolution path, core journals, productive countries, collaboration networks, and research frontiers from a macro level. Second, bibliometric analysis has been widely applied in public health, nursing, and medical informatics in recent years, demonstrating its value in synthesizing large-scale literature. As Kokol et al. (99) noted, bibliometrics allows researchers to analyze large numbers of publications and their production patterns at both macro and micro levels. Third, the intersection of digital health interventions and health equity has rarely been examined using bibliometric methods, making this study a novel contribution. To address the inherent limitations of purely quantitative bibliometrics—such as the assumption that “high frequency equals importance”—we follow the Synthetic Knowledge Synthesis (SKS) approach proposed by (24, 100), which triangulates bibliometrics, bibliometric mapping, and content analysis to achieve both quantitative description and qualitative interpretation (25).

Three bibliometric tools were used in a complementary workflow. **Bibliometrix** (R package) served as the data integration layer—cleaning, deduplicating, and standardizing fields—and generated basic statistical indicators (annual trends, collaboration intensity). **SciExplorer** served as the visualization layer—producing geographic distribution maps and journal ranking visualizations. **CiteSpace** (v.6.4.R1) served as the deep mining layer—constructing keyword co-occurrence networks, detecting burst terms, and calculating node centrality indicators. This three-layer workflow (integration → visualization → mining) ensures that each tool contributes uniquely while maintaining analytical coherence (26).

Bibliometrix (R package) was used for data integration and basic statistical analysis. It cleaned, deduplicated, and standardized fields of the merged literature. It generated annual publication trends, author collaboration intensity, country/region collaboration networks, and other basic indicators. This provided a standardized data base for deeper analysis. SciExplorer was used for visualization of geographic and journal distributions. Its spatial data visualization module drew global publication volume maps. It also generated rankings of core journal publication volumes and annual journal publication trends. This revealed platform characteristics and dynamic evolution of knowledge dissemination in the field. CiteSpace (v.6.4.R1) was used for in-depth mining of knowledge structure and research frontiers. It was the core analytical tool of this study. Its specific methods included: constructing keyword co-occurrence networks and cluster maps, using the LLR algorithm to identify research hotspots and calculate cluster silhouette values and modularity Q values; drawing interdisciplinary maps and journal dual-map overlays to show knowledge flow paths across fields; generating author collaboration network maps and calculating node centrality indicators (betweenness centrality, closeness centrality, PageRank, etc.) to identify core authors and bridge scholars; and detecting research frontiers and thematic evolution trends through burst detection.

“For the purposes of this study, ‘structural bias’ is operationally defined as systematic, non-random patterns in knowledge production that reflect unequal distribution of research capacity, attention, and influence across geographic regions, thematic areas, and collaboration networks. These biases are empirically identified through bibliometric indicators:

- Geographic bias is measured by the concentration of publication frequencies across countries/regions, with the United States accounting for >54% of the top ten countries’ total frequencies and the near-absence of African countries in the dataset (92).
- Thematic bias is identified through keyword clustering analysis, where the absence of ‘health equity’ as an independent high-frequency cluster, and the lack of keywords directly referencing equity dimensions (e.g., ‘low-income population,’ ‘rural population’), indicates systematic under-attention to equity as a primary research focus.
- Power-structural bias is inferred from centrality measures (betweenness centrality, PageRank) in the author collaboration network, where a small number of ‘knowledge gatekeepers’—predominantly from high-income, English-speaking countries—occupy bridging positions that control knowledge flow across sub-fields.

These indicators do not directly measure causality or intentional exclusion. Rather, they reveal patterned asymmetries in knowledge production that, when interpreted through the theoretical lens of science and technology studies and health equity frameworks, suggest structural rather than random or benign distribution of research attention and influence.”

## 3 Results

Three bibliometric tools were used in a complementary workflow. Bibliometrix (R package) served as the data integration layer—cleaning, deduplicating, and standardizing fields—and generated basic statistical indicators (annual trends, collaboration intensity). SciExplorer served as the visualization layer—producing geographic distribution maps and journal ranking visualizations. CiteSpace (v.6.4.R1) served as the deep mining layer—constructing keyword co-occurrence networks, detecting burst terms, and calculating node centrality indicators. This three-layer workflow (integration → visualization → mining) ensures that each tool contributes uniquely while maintaining analytical coherence.

### 3.1 General overview and literature characteristics

Table 2 summarises the main characteristics of the included documents. Between 2015 and 2025, this study included 4,677 documents from 1,460 academic journals. The annual growth rate was as high as 33.18%. The average age of documents was only 3.6 years. This indicates that the field is not slowly accumulating but has exploded exponentially in the last five years. This rapid growth is closely related to the rapid iteration of digital health technologies (such as wearables and mobile health apps) and increased policy attention to health equity globally. Meanwhile, the average citation per document was 15.05. Total references were nearly 170,000 (about 36.3 references per document). This reflects that the field's outputs have good academic influence and a very wide range of knowledge integration. Researchers need to master multidisciplinary foundational literature from biomedicine, behavioral science, information engineering, and sociology.

**Table 2:** Summary statistics of the study.

Description	Results
MAIN INFORMATION ABOUT DATA	
Timespan	2015:2025
Sources (Journals, Books, etc)	1460
Documents	4677
Annual Growth Rate %	33.18
Document Average Age	3.6
Average citations per doc	15.05
References	169876
DOCUMENT CONTENTS	
Keywords Plus (ID)	4971
Author's Keywords (DE)	9302
AUTHORS	
Authors	24881
Authors of single-authored docs	106
AUTHORS COLLABORATION	
Single-authored docs	115
Co-Authors per Doc	6.88
International co-authorships %	27.3
DOCUMENT TYPES	
Article	3,526
article; book chapter	1
article; data paper	2
article; early access	54
article; proceedings paper	5
article; retracted publication	3
journal article	190

Second, the number of authors is large (24,881), but single-author documents account for only 2.5%. The average number of co-authors per document is 6.88. International co-authorships (authors from two or more countries) account for 27.3%. The total frequency of appearances of authors from different countries (about 18,000) is much larger than the total number of documents (4,677). On average, each paper involves 3.85 countries. This reflects the highly transnational collaborative nature of research in this field. This pattern is not accidental. Reasons may include that digital health interventions involve technology development, clinical validation, behavioral evaluation, and equity analysis, so a single discipline or institution cannot complete them independently. Also, data collection in low- and middle-income countries often requires collaboration with teams from high-income countries, thus increasing the proportion of international cooperation. However, high collaboration may also lead to resources concentrating in a few core teams, forming “knowledge elite circles,” which may further reduce the visibility of marginalized groups and marginalized issues.

Notably, among the 4,677 documents, there were 9,302 distinct author keywords. Only 341 of these appeared more than five times. This reflects a lack of a unified terminology system in the field. Keywords Plus are automatically extracted from document references and are highly standardized and consistent across documents. Author keywords are freely chosen by researchers. Their high dispersion indicates that although research topics are highly concentrated, core concepts (such as “digital health” vs. “eHealth,” “health equity” vs. “health inequality”) have not yet formed a unified terminology system. This conceptual fragmentation directly hinders systematic evidence synthesis. In systematic reviews, keyword heterogeneity may lead to missed retrieval. In policy translation, vague terminology weakens the operability of guidelines. Therefore, the field currently enjoys the benefits of rapid growth but also faces the structural challenge of “quantity increase with quality dispersion.” Future knowledge accumulation requires not only more research but also systematic adjustments in concept standardization, inclusion in collaborative models, and depth of interdisciplinary integration.

### 3.2 Time series characteristics

**Figure 2:**
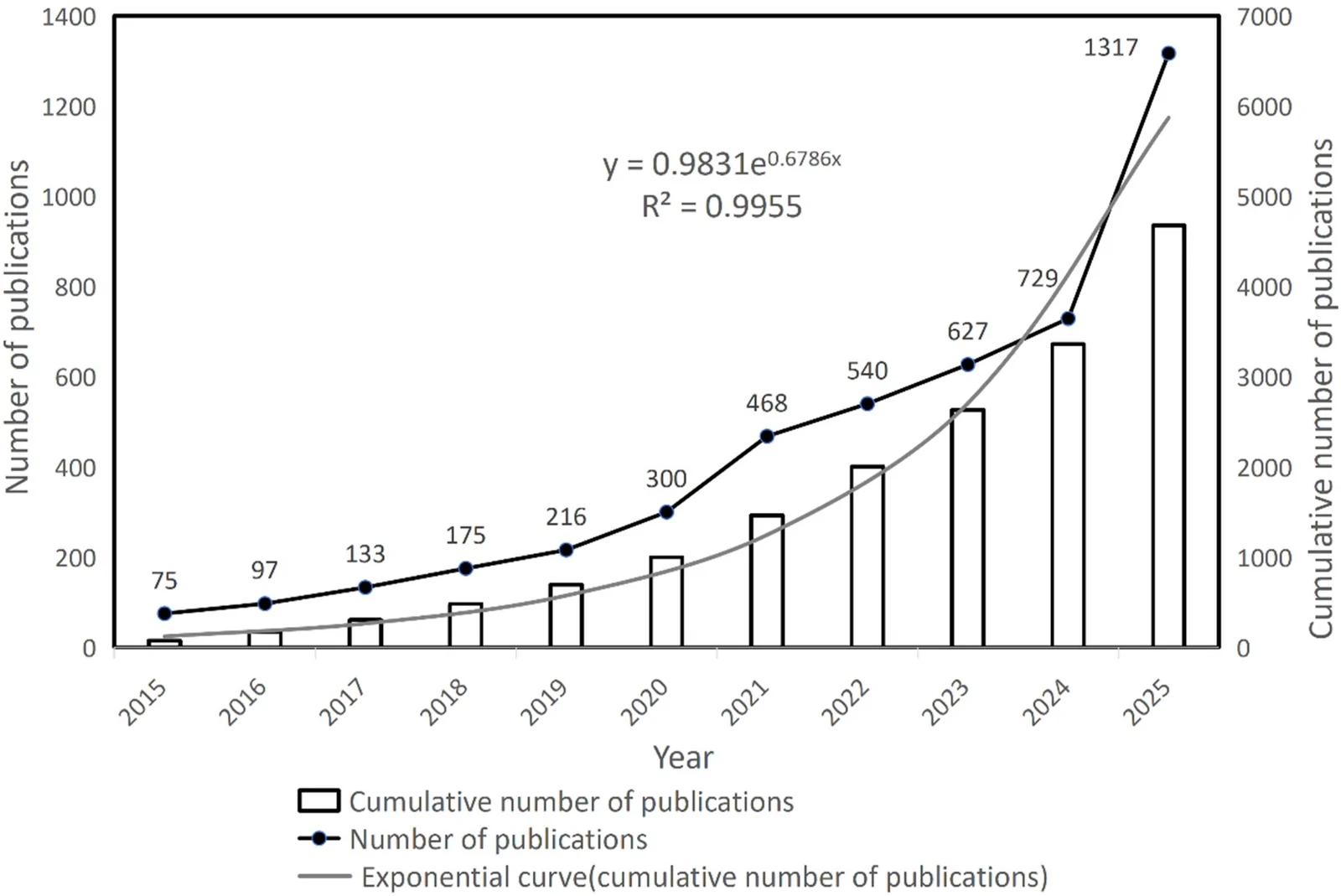
Annual publication trend.

Figure 2 shows the annual publication trend from 2015 to 2025. In 2015, the annual number of publications was 75. Then it increased year by year: 97 in 2016, 133 in 2017, 175 in 2018, 216 in 2019, 300 in 2020, 468 in 2021, 540 in 2022, 627 in 2023, 729 in 2024, and 1,317 in 2025. Over the ten years, the annual number grew about 16.6 times. The cumulative number grew from 75 to 4,677, about 61.4 times. Looking at the curve shape, growth was slow from 2015 to 2017. The slope gradually increased from 2018 onward. It became significantly steeper after 2021 and peaked in 2025, showing a typical accelerating upward trend.

First, the growth rate accelerated over time, with clear acceleration characteristics. Taking three-year stages to calculate average annual increments: from 2015 to 2017, annual publications increased from 75 to 133, average annual increment about 29. From 2018 to 2020, from 175 to 300, average annual increment about 42. From 2021 to 2025, from 468 to 1,317, the average annual increment jumped to about 170. Especially in 2025 alone, the increment was 588, exceeding the sum of increments from 2015 to 2019 (141). This shows that knowledge production in this field has moved from slow accumulation in the early stage to rapid expansion in the later stage, and the expansion speed itself is accelerating.

Second, the growth trajectory can be divided into three logically coherent sub-stages (27). 2015–2017 was a low-speed accumulation period, with annual publications increasing from 75 to 133 and cumulative total reaching 305. Research in this stage mainly focused on proof-of-concept, small-scale pilots, and theoretical framework construction. There was not yet a broad research consensus, and the academic output base was low with small increments. 2018–2020 was a steady rise period, with annual publications increasing from 175 to 300 and cumulative total approaching 1,000 (996). The acceleration in this period coincided in time with the spread of smartphones, commercialization of wearables, and attention to digital health by the World Health Organization and other institutions. This suggests that external technology supply and policy agendas began to translate into stable academic output. 2021–2025 was an explosive expansion period, with annual publications jumping from 468 to 1,317 and cumulative total expanding from 1,464 to 4,677. Particularly in 2025, the annual publication volume reached 1,317, an 80.7% increase over the previous year, the highest annual growth rate in the period. This explosion resulted from a positive feedback loop formed by multiple forces: earlier accumulated technology, policy, academic inertia, and the telehealth demand triggered by the COVID-19 pandemic. Each new study can inspire subsequent studies, and each technological breakthrough opens new empirical directions, thus maintaining and strengthening the accelerating growth.

Third, the relationship between flow (annual publications) and stock (cumulative publications) has fundamentally changed. In 2015, both flow and stock were 75, a 1:1 ratio. By 2020, flow was 300 and stock was 996, a ratio of about 1:3.3. By 2025, flow was 1,317 and stock was 4,677, a ratio of about 1:3.6. More importantly, the absolute value of flow has jumped from less than 100 per year to more than 1,000 per year. This means that the amount of literature added in 2025 alone is about 1.9 times the total of the five years from 2015 to 2019. For researchers, the total of 4,677 documents is already beyond the capacity of traditional narrative reviews or manual close reading. The annual addition of over 1,000 documents makes “continuous tracking” impossible. Therefore, the accelerating growth revealed by the trend chart is not just a quantitative phenomenon but also a methodological warning. The field must shift from a manual induction paradigm to an automated knowledge graph analysis based on scientometrics. Otherwise, it will face the paradox of “more literature, less global insight.”

### 3.3 Interdisciplinary patterns

**Figure 3:**
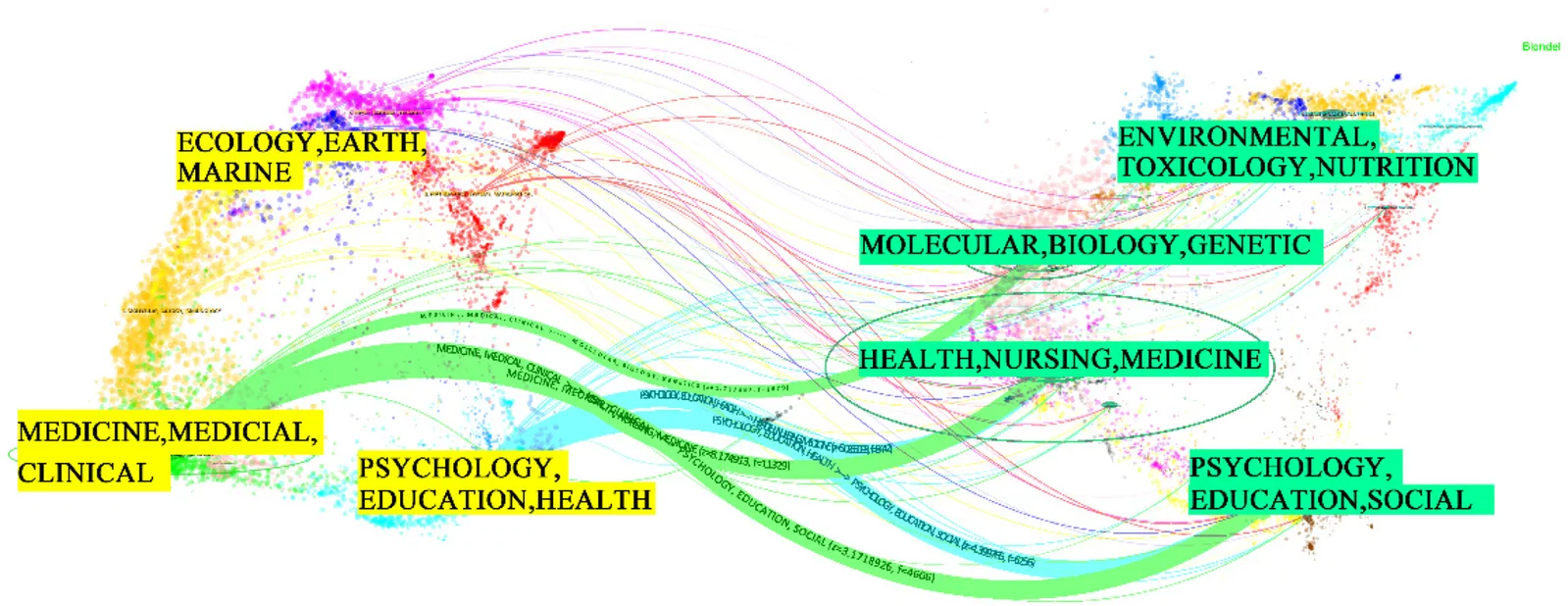
Interdisciplinary network map.

Figure 3 presents the interdisciplinary network map based on journal dual-map overlay. This figure shows the journal dual-map overlay analysis result in bibliometrics. It presents knowledge flow paths between citing journals and cited journals through different colored blocks and connecting lines. Based on the subject labels extracted from the figure and their combinations, the knowledge groups appearing in the figure can be divided into the following main disciplinary blocks: Earth and Environmental Sciences block, including ecology, earth sciences, environmental science, and oceanography; Basic Biomedical block, including toxicology, nutrition, and molecular biology & genetics; Clinical and Health Sciences block, including health, nursing, medicine, and clinical medicine; Behavioral and Social Sciences block, including psychology, education, and social sciences.

From the perspective of the interdisciplinary nature of this study's topic (digital health intervention, patient engagement/adherence, health equity), there are multi-level knowledge connections between the above blocks and this study. The Clinical and Health Sciences block is the most direct knowledge source. The evaluation of digital health intervention effects, clinical measurement of patient adherence, and remote monitoring in chronic disease management all directly rely on evidence systems from medicine, nursing, and clinical research. The Behavioral and Social Sciences block is also closely related. The theoretical basis of patient engagement, intervention design for health behavior change, social determinants of the digital divide, and the educational aspects of health equity are all rooted in the research accumulation of psychology, education, and sociology. The appearance of the Basic Biomedical block can also be explained academically. Some digital health interventions target health problems related to nutrition metabolism, toxic exposure, or genetic susceptibility. Therefore, when discussing intervention mechanisms or outcome indicators, they may cite literature from toxicology, nutrition, and molecular biology. Content about environmental justice in health equity issues may also involve unequal distribution of environmental exposures. The connection with the Earth and Environmental Sciences block is relatively indirect. Some digital health applications are used for environmental health monitoring, climate-related health interventions, or agricultural nutrition promotion, possibly citing methodological literature from ecology or earth sciences. Also, the broadness of journal classification may cause this block to appear in the overlay.

The visual proportions and connection strengths of the blocks in the figure reflect the actual magnitude of knowledge flow in the citation network. For this study, the clinical health block and behavioral/social sciences block should dominate, while the earth/environment block and basic biomedical block should have limited proportions. Overall, the figure confirms at a macro level the interdisciplinary nature of this research field. Its knowledge inputs span multiple disciplinary levels from basic biomedicine to clinical practice, from individual behavior to social structure. The coexistence of different disciplinary blocks reflects the breadth of knowledge integration in this field.

### 3.4 Geographic distribution characteristics

**Figure 4:**
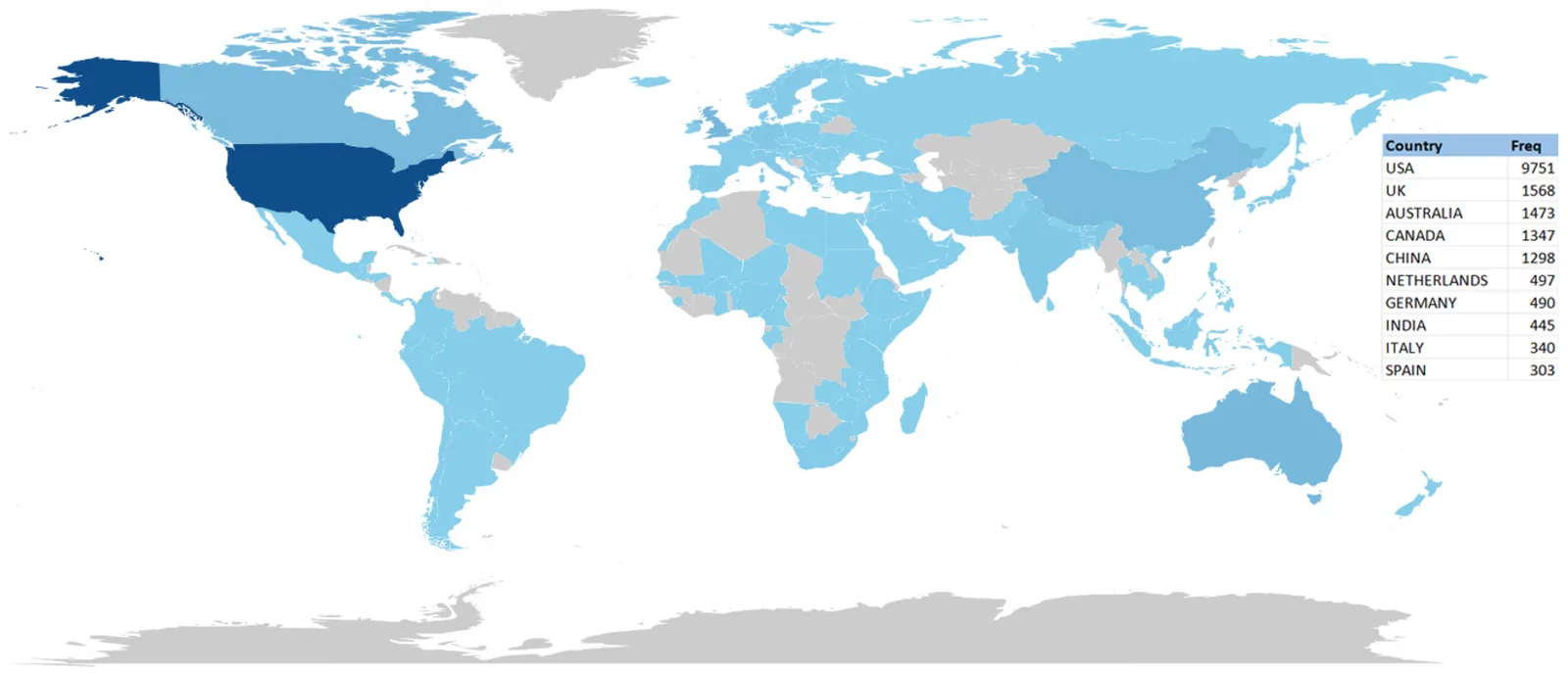
Frequency of participation by country/region from 2020 to 2025.

Figure 4 illustrates the frequency of participation by country/region from 2020 to 2025. Based on the publication frequency statistics of each country/region, the United States ranked first with 9,751 occurrences, accounting for more than 54% of the total frequency of the top ten countries (about 18,000). This was 6.2 times that of the second-ranked United Kingdom (1,568). The United Kingdom, Australia (1,473), Canada (1,347), and China (1,298) formed the second tier. Their frequencies were concentrated between 1,300 and 1,600, with small differences. The third tier included the Netherlands (497), Germany (490), India (445), Italy (340), and Spain (303). Their frequencies were significantly lower than the top five. It should be noted that the frequency counts the number of appearances of all author affiliations in each document. Therefore, one internationally co-authored paper contributes one frequency to each country involved. The total frequency (about 18,000) is much larger than the total number of documents (4,677), which confirms the high international collaboration rate (27.3%) in this field. In terms of distribution pattern, the United States shows an absolute “unipolar” lead. The second-tier countries compete closely. The third tier decays rapidly.

This geographic distribution pattern reveals multiple structural implications. First, English-speaking countries (USA, UK, Australia, Canada) occupied the top four positions, together accounting for about 14,000 frequencies, or 78% of the top ten total (28). This high concentration may partly stem from the indexing preferences of the three databases (Scopus, WoS, PubMed) for English-language journals, but it also truly reflects the first-mover advantages of English-speaking countries in research funding, academic infrastructure, and international collaboration networks (27). For non-English-speaking countries, even if their research is published in English journals, they may face additional language barriers and publication costs, inhibiting some high-quality research production (21). Second, China is the only non-English-speaking country to enter the top five, with a frequency of 1,298, at the same level as Australia and Canada. This indicates that China has strong research output capacity in this field (29). However, given China's population size, total number of researchers, and GDP investment, this frequency still has room for improvement. Moreover, Chinese scholars participate more in domestic collaborations than lead international networks. Third, India, another major developing country, ranked only eighth (445), about one-third of China's. This is related to differences between the two countries in digital infrastructure, Internet penetration, and investment in public health research (30). More worryingly, the marginalization of low- and middle-income countries in global research output is ironic because it mirrors the “digital divide” that the health equity topic focuses on (31). Digital health interventions aim to reduce health inequalities, but the related research itself is dominated by high-income countries, lacking local evidence from low-income countries (32). Fourth, although several European countries appear on the list (UK, Netherlands, Germany, Italy, Spain), except for the UK, their frequencies are below 500, showing a pattern of “fragmented but not low total” (Europe total about 3,300), while the USA alone reached 9,751. This reflects the fragmentation of the European research system and the advantage of centralized investment at the national level in the USA (33). In summary, the geographic pattern of research output in this field is “one superpower and multiple strong countries,” dominated by English-speaking countries, with China catching up quickly, but the Global South is severely underrepresented (30). This imbalance may weaken the generalizability and equity of digital health interventions across different cultural and economic backgrounds, suggesting that future research funding and policy should prioritize building local research capacity in low- and middle-income countries. **Country Collaboration Network Analysis.** To complement the geographic frequency analysis, we constructed a country-level collaboration network based on co-authorship relationships. The network revealed a “one superpower and multiple strong nodes” pattern, with the United States serving as the central hub (degree centrality = 0.87), bridging collaborations between European and Asian countries. The UK, Australia, and Canada functioned as secondary hubs with high betweenness centrality, facilitating knowledge flow between the US and other regions. China, despite its high publication volume, exhibited low betweenness centrality (0.12), indicating that Chinese scholars primarily collaborate domestically or with the US rather than serving as bridges for broader international networks. This structural pattern reinforces the geographic concentration of knowledge production identified in our frequency analysis (25).

### 3.5 Journal publication characteristics

**Figure 5:**
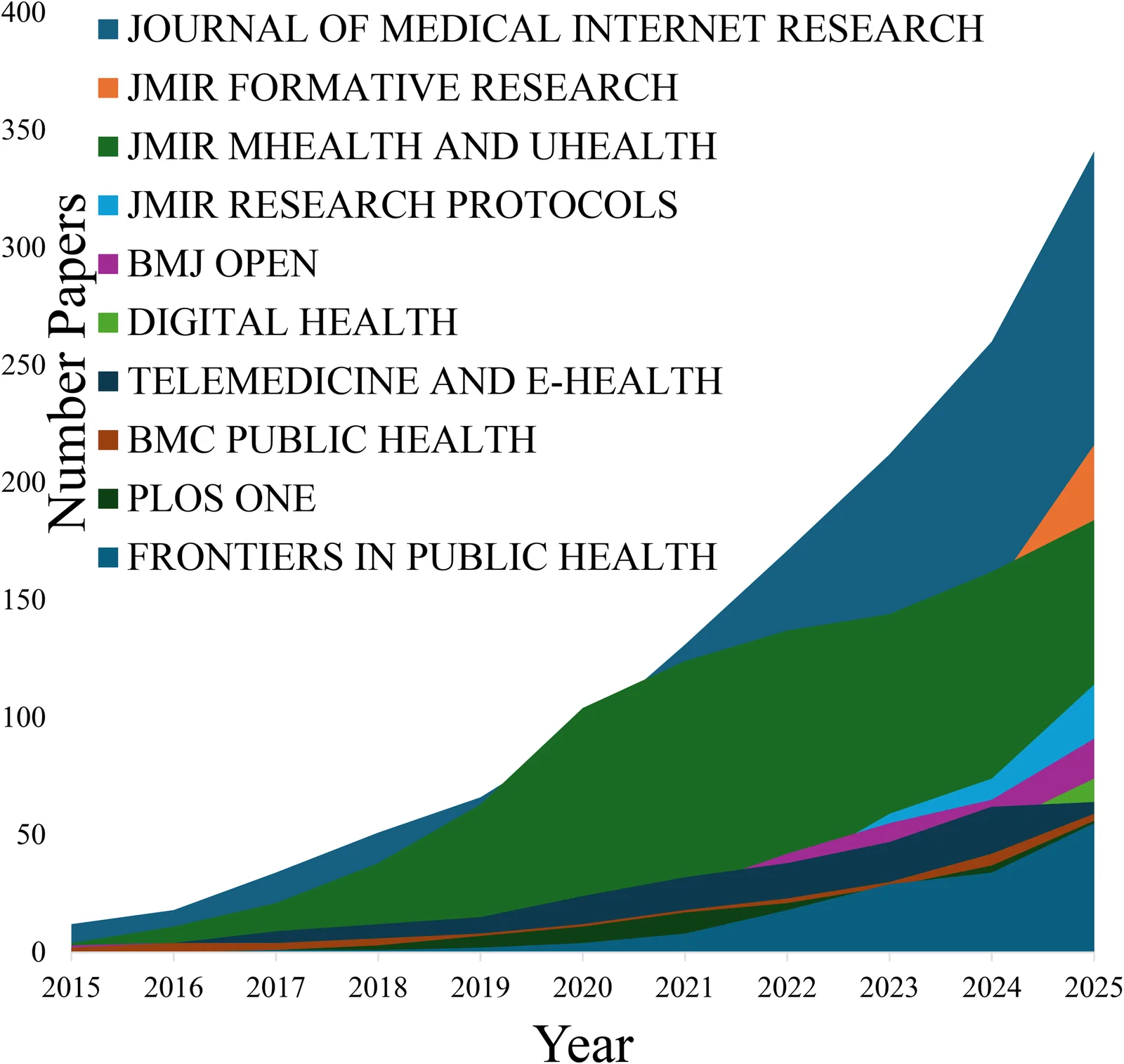
Annual publication trends of top 10 journals.

Figure 5 displays the annual publication trends of the top 10 journals. Among the top ten journals, the JMIR series dominated. The Journal of Medical Internet Research ranked first with 341 articles. JMIR Formative Research (216), JMIR mHealth and uHealth (184), and JMIR Research Protocols (114) ranked second, third, and fourth respectively. Together, they published 855 articles, accounting for 58.8% of the top ten total (1,454). Other journals included BMJ Open (91), Digital Health (74), Telemedicine and e-Health (64), BMC Public Health (59), PLOS ONE (56), and Frontiers in Public Health (55).

Table 3 lists the top 10 journals by publication volume and their impact factors. In terms of quartiles, there were five Q1 journals (two of the JMIR series, Digital Health, BMC Public Health, Frontiers in Public Health), two Q2 journals (BMJ Open, PLOS ONE), and three Q3 journals (JMIR Formative Research, JMIR Research Protocols, Telemedicine and e-Health). The highest 5-year impact factor was for JMIR mHealth and uHealth (6.2), followed by the Journal of Medical Internet Research (6.0). The lowest was for JMIR Research Protocols (1.5).

**Table 3:** Top 10 journals by publication volume in this field and their impact factors.

Journal	Articles	Quartile	IF (in recet 5 years）
JOURNAL OF MEDICAL INTERNET RESEARCH	341	Q1	6
JMIR FORMATIVE RESEARCH	216	Q3	2.1
JMIR MHEALTH AND UHEALTH	184	Q1	6.2
JMIR RESEARCH PROTOCOLS	114	Q3	1.5
BMJ OPEN	91	Q2	2.3
DIGITAL HEALTH	74	Q1	3.3
TELEMEDICINE AND E-HEALTH	64	Q3	2
BMC PUBLIC HEALTH	59	Q1	3.6
PLOS ONE	56	Q2	2.6
FRONTIERS IN PUBLIC HEALTH	55	Q1	3.4

This journal distribution pattern reveals several core features of knowledge dissemination in this field. First, the absolute dominance of the JMIR series journals (four journals, accounting for nearly 60% of the top ten total) indicates that research on digital health interventions, patient engagement, and health equity is highly concentrated in this specialized journal cluster, forming an “academic community” publishing ecosystem (34). The JMIR series is known for open access and rapid peer review, making it particularly suitable for interdisciplinary and fast-evolving topics like digital health (35). Second, the coexistence of Q1 and Q3 journals is noteworthy. High-impact journals (such as JMIR mHealth and uHealth and the Journal of Medical Internet Research) mainly publish original, methodologically rigorous studies. Q3 journals (such as JMIR Formative Research and JMIR Research Protocols) focus on feasibility studies, protocol designs, and preliminary data. This stratification facilitates the dissemination of the full research lifecycle, from early exploration to mature validation (36). Third, the inclusion of comprehensive or public health journals such as BMJ Open, BMC Public Health, PLOS ONE, and Frontiers in Public Health shows that this research topic has moved beyond pure digital technology and is deeply integrating with public health, epidemiology, and health services research (37). Finally, none of the top ten journals are traditional top-tier medical journals (such as NEJM, The Lancet, JAMA). This suggests that the field is still in a “periphery-to-center” transition period in terms of interdisciplinary status (38). It has not yet fully penetrated mainstream clinical medicine journals, but this also leaves room for future research outputs to move to higher-impact platforms (39). **Institutional Collaboration Network Analysis** (40). At the institutional level, the collaboration network comprised 3,842 unique institutions. The top five institutions by publication volume were Harvard University (*n* = 187), University of California San Francisco (*n* = 156), University of Toronto (*n* = 142), University College London (*n* = 128), and Johns Hopkins University (*n* = 119). Notably, all top-ten institutions were located in high-income English-speaking countries. Network analysis revealed that knowledge production is concentrated in a small number of elite research universities, which function as institutional knowledge hubs. Institutions from LMICs were predominantly positioned at the network periphery, with limited connections to core hubs, mirroring the country-level center-periphery structure.

Based on the stacked annual publication volumes of 10 core journals from 2015 to 2025, the journal publication pattern in this field shows three stage-specific characteristics. From 2015 to 2017, the Journal of Medical Internet Research and JMIR mHealth and uHealth were the main contributors. Other journals published very few articles. From 2018 to 2020, the JMIR series continued to dominate. Meanwhile, JMIR Formative Research and JMIR Research Protocols began publishing. Journals such as BMJ Open, Digital Health, and Telemedicine and e-Health gradually appeared. From 2021 to 2025, both the number of journals and publication volumes expanded rapidly. The combined annual publication volume of the four JMIR journals increased from about 296 in 2021 to 855 in 2025. At the same time, the publication volumes of public health comprehensive journals such as BMC Public Health, PLOS ONE, and Frontiers in Public Health rose significantly, reaching 59, 56, and 55 in 2025 respectively. Overall, the JMIR series always held an absolute advantage, but the share of other journals increased in the later period.

This stacked area chart reveals the dynamic evolution of knowledge dissemination platforms in the field. First, the JMIR series journals (especially the Journal of Medical Internet Research and JMIR mHealth and uHealth) are not only the largest publishers but also the core venues in the early stage of the field (41). Their early monopoly (almost all articles from 2015 to 2017 came from these two journals) indicates that digital health intervention research initially relied heavily on specialized open-access journals (42). Second, the sharp increase in publications from JMIR Formative Research and JMIR Research Protocols after 2021 reflects the differentiation of research types within the field (41). The former focuses on formative evaluations and feasibility studies, while the latter focuses on study protocol registration. This indicates that the field has moved from mere result dissemination to full research lifecycle management. Third, the steady growth of comprehensive OA journals such as BMJ Open, BMC Public Health, PLOS ONE, and Frontiers in Public Health shows that digital health and health equity research is being accepted by the broader public health academic community (43). The research topic is beginning to integrate into mainstream public health issues. Fourth, Telemedicine and e-Health, a traditional telemedicine journal, grew relatively slowly (64 articles in 2025), suggesting that the field may be transitioning from broad telemedicine to more specialized digital health interventions (44). Overall, the stacked area chart shows an evolution from “monopoly by a single specialized journal” to “multiple journal types jointly carrying the field” (42), reflecting the field's disciplinary maturity and diffusion of academic influence.

### 3.6 Research collaboration characteristics

**Figure 6:**
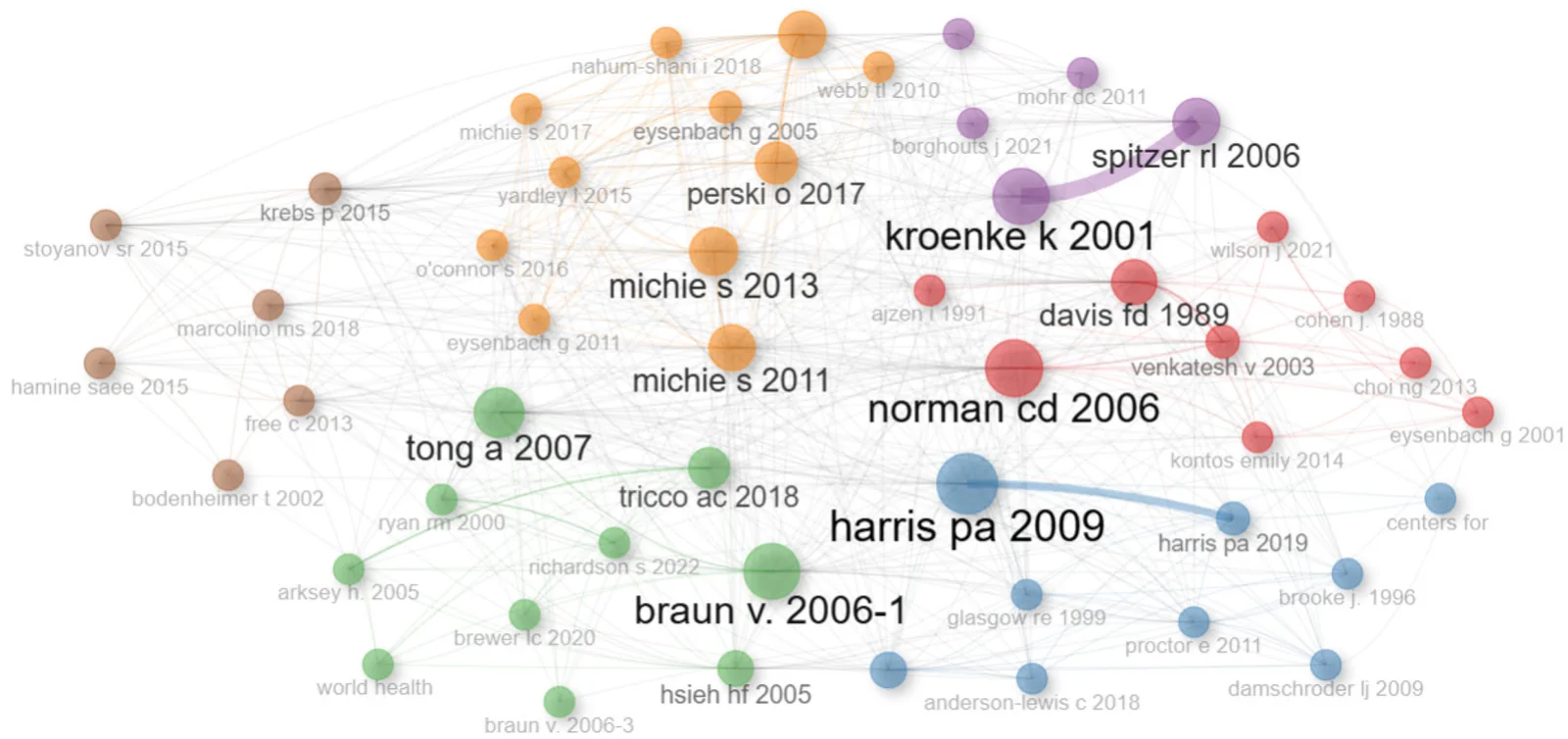
Author collaboration network map.

Figure 6 shows the author collaboration network map. The figure lists key nodes in the author collaboration or co-citation network of this field, including nahum-shami i 2018, eysenbach g 2005, perski o 2017, kroenke k 2001, michie s 2013, davis f 1989, norman cd 2006, tricco ac 2018, harris pa 2009, braun v. 2006-1, hsieh nf 2005, andersson-lewis c 2018, spitzer rl 2006, wilson j 2021, cohen j 1998, chong n 2013, eysenbach g 2001, brooke j 1996, damschroder lj 2009, etc. These authors and years span from 1989 to 2021. Early works (such as davis 1989, cohen 1998) relate to technology acceptance models and effect size statistics. Recent works (such as wilson 2021, nahum-shami 2018) focus on digital interventions and adaptive study designs.

These nodes reveal the knowledge base and academic heritage of the field. First, the appearance of behavioral scientists such as michie (2013) (45) and perski (2017) (46)indicates that behavior change theories (e.g., COM-B model, person-based design) are important theoretical pillars of this field. Davis (46, 47) and his Technology Acceptance Model (TAM) provide a classic framework for user adoption of digital health interventions. Second, eysenbach (2001, 2005) (48), as an early definer of the “eHealth” concept, his node position reflects the foundational contributions to digital health research. Third, scholars such as kroenke (2001) (49)and spitzer (2006) are associated with the development of psychometric tools such as the Patient Health Questionnaire (PHQ-9). This indicates that patient-reported outcomes occupy a core position in adherence and health equity research. Fourth, harris (2009) and damschroder (2009) (50) are closely linked to implementation science frameworks (such as CFIR, RE-AIM), reflecting the field's expansion from pure efficacy evaluation to implementation and sustainability assessment (33). Finally, braun (2006) (51) and tricco (2018) (52) are related to qualitative research and systematic review methodologies, suggesting that methodological diversity is a notable feature of this field. Overall, the node composition shows that the field deeply integrates behavioral science, informatics, implementation science, and clinical measurement, forming a multi-disciplinary academic collaboration network.

Table 4 presents node centrality indicators (betweenness, closeness, PageRank) in the scientific collaboration network. This table shows the centrality indicators of important nodes in the author collaboration or co-citation network, including betweenness centrality, closeness centrality, and PageRank. According to cluster divisions, the nodes are distributed across six clusters. Cluster 1 includes norman cd 2006 (betweenness 128.645, closeness 0.043, PageRank 0.032), davis fd 1989 (46.098, 0.040, 0.032), etc. Norman and Davis have high betweenness centrality, indicating they act as bridges between different research groups. Cluster 2 includes harris pa 2009 (69.893, 0.038, 0.041), braun v. 2006-2 (26.644, 0.035, 0.015), etc. Harris pa 2009 has the highest PageRank (0.041), indicating it is a core authoritative node in that cluster. Cluster 3 includes braun v. 2006-1 (103.228, 0.043, 0.028), tong a 2007 (60.831, 0.040, 0.032), etc. Braun v. 2006-1 has extremely high betweenness centrality, playing an important knowledge transfer role. Cluster 4 includes kroenke k 2001 (52.154, 0.038, 0.039), spitzer rl 2006 (18.985, 0.035, 0.035). Both have high PageRank and betweenness centrality. Cluster 5 includes michie s 2011 (63.041, 0.040, 0.028), yardley l 2016 (35.683, 0.039, 0.042). Yardley l 2016 has the highest PageRank (0.042). Michie s 2013 has a betweenness centrality of 34.44. Cluster 6 includes free c 2013 (24.753, 0.035, 0.018), krebs p 2015 (51.272, 0.041, 0.016). Krebs p 2015 has high betweenness centrality.

**Table 4:** Node centrality indicators in the scientific collaboration network.

Node	Cluster	Betweenness	Closeness	PageRank
norman cd 2006	1	128.645	0.043	0.032
davis fd 1989	1	46.098	0.04	0.032
venkatesh v 2003	1	8.007	0.033	0.028
cohen j. 1988	1	0.475	0.029	0.009
kontos emily 2014	1	3.629	0.029	0.017
ajzen i 1991	1	8.065	0.035	0.012
choi ng 2013	1	0	0.025	0.018
eysenbach g 2001	1	3.151	0.029	0.013
wilson j 2021	1	2.005	0.029	0.014
harris pa 2009	2	69.893	0.038	0.041
braun v. 2006-2	2	26.644	0.035	0.015
harris pa 2019	2	0.797	0.033	0.031
proctor e 2011	2	14.6	0.034	0.017
glasgow re 1999	2	2.618	0.034	0.019
damschroder lj 2009	2	4.225	0.033	0.015
brooke j. 1996	2	1.921	0.032	0.009
anderson-lewis c 2018	2	4.608	0.032	0.011
centers for	2	0.02	0.027	0.005
braun v. 2006-1	3	103.228	0.043	0.028
tong a 2007	3	60.831	0.04	0.032
tricco ac 2018	3	12.562	0.034	0.027
hsieh hf 2005	3	23.327	0.037	0.021
ryan rm 2000	3	2.474	0.032	0.011
richardson s 2022	3	3.439	0.031	0.018
arksey h. 2005	3	4.562	0.031	0.018
braun v. 2006-3	3	0.556	0.028	0.01
world health	3	0	0.027	0.01
brewer lc 2020	3	1.806	0.03	0.01
kroenke k 2001	4	52.154	0.038	0.039
spitzer rl 2006	4	18.985	0.035	0.035
borghouts j 2021	4	18.267	0.033	0.015
mohr dc 2011	4	4.776	0.033	0.012
baumel a 2019	4	7.979	0.033	0.016
michie s 2011	5	63.041	0.04	0.028
michie s 2013	5	34.44	0.036	0.036
yardley l 2016	5	35.683	0.039	0.042
perski o 2017	5	29.101	0.036	0.035
eysenbach g 2005	5	20.039	0.035	0.023
webb tl 2010	5	8.357	0.034	0.018
o'connor s 2016	5	22.377	0.035	0.018
eysenbach g 2011	5	5.586	0.033	0.013
michie s 2017	5	1.328	0.033	0.025
nahum-shani i 2018	5	3.595	0.033	0.015
yardley l 2015	5	4.064	0.034	0.022
free c 2013	6	24.753	0.035	0.018
krebs p 2015	6	51.272	0.041	0.016
stoyanov sr 2015	6	14.436	0.036	0.011
marcolino ms 2018	6	8.188	0.034	0.015
hamine saee 2015	6	2.395	0.031	0.014
bodenheimer t 2002	6	0	0.023	0.008

Betweenness centrality values are raw (not normalized) and are only used for comparing nodes within the network.

These centrality indicators reveal the structural roles of different scholars in the academic collaboration network. First, nodes with high betweenness centrality (such as norman cd 2006, braun v. 2006-1, harris pa 2009, krebs p 2015) act as “knowledge bridges.” They connect different research groups or clusters and facilitate the flow of ideas across fields (53). For example, norman is often associated with health informatics, and braun with qualitative research methods. Their high betweenness centrality indicates that methodologists play key roles in integrating different research directions. Second, nodes with high PageRank (such as yardley l 2016, harris pa 2009, kroenke k 2001, spitzer rl 2006) are “authority cores” within their clusters. Their research outputs are widely cited or they have extensive collaborations (54). Yardley l 2016 is highly influential in behavioral intervention design (55). Harris pa 2009 is known for the REDCap tool (56). Kroenke and spitzer are authorities in mental health measurement due to the PHQ-9 scale (8, 11). Third, closeness centrality values are generally similar (0.023–0.043), indicating good network connectivity. Most nodes can connect to other nodes relatively quickly. It is worth noting that different year versions of the same author (e.g., michie s 2011, 2013, 2017; braun v. 2006) (51) appear in different clusters. This reflects changes in that scholar's research direction or collaboration patterns over time. It also suggests that the network construction may treat different years of the same author as separate nodes. Overall, the centrality structure of this network shows that the field has formed a multi-center collaboration pattern with methodologists (qualitative, implementation science), theory builders (behavior change, technology acceptance), and tool developers (measurement scales, data platforms) as cores.

### 3.7 Keyword clustering

**Figure 7:**
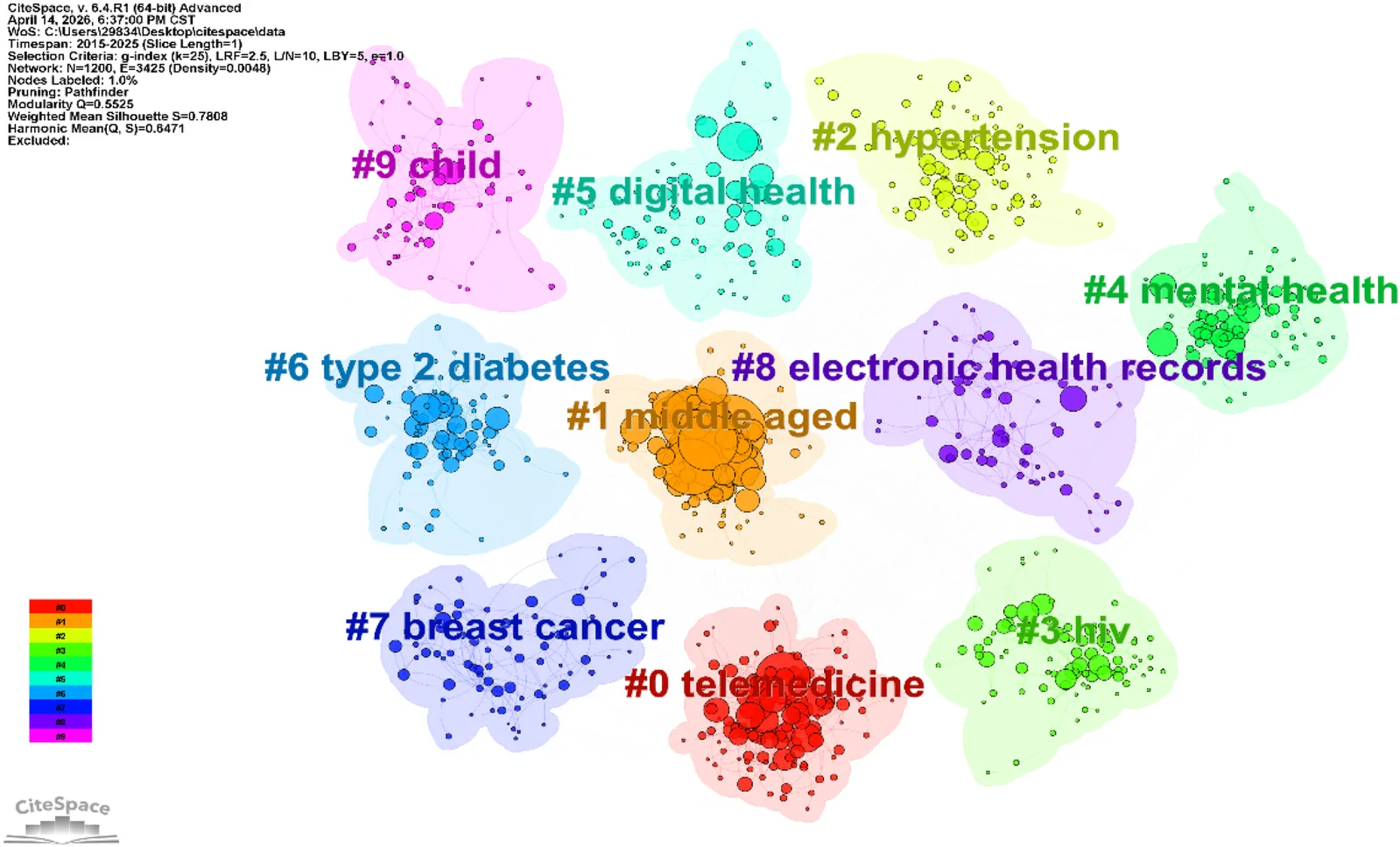
Keyword clustering map.

Figure 7 is the keyword clustering map generated by CiteSpace. This figure is a keyword clustering map generated by CiteSpace. Network parameters show *N* = 1200 nodes, E = 3425 links, network density of 0.0048. The analysis used g-index (k = 2) and Pathfinder pruning. The modularity Q value of the clustering was 0.5525. The weighted mean silhouette value S was 0.7868. The harmonic mean (Q,S) was 0.8471. The figure identifies 10 main clusters: #0 telemedicine, #1 middle aged, #2 hypertension, #3 hiv, #4 mental health, #5 digital health, #6 type 2 diabetes, #7 breast cancer, #8 electronic health records, and #9 child. Among these, #0 telemedicine, #5 digital health, and #9 child are larger or located in the core of the map.

This cluster structure reveals the diverse thematic layout of research in this field. First, clusters #0 telemedicine and #5 digital health dominate, indicating that telemedicine and digital health are the core themes of the entire knowledge network. The research surrounding them is the richest. Second, chronic disease management is an important application context. This is shown by clusters #2 hypertension, #6 type 2 diabetes, and #7 breast cancer. Digital health interventions are mainly concentrated in these disease areas that require long-term management and are suitable for remote monitoring. Third, clusters #4 mental health and #9 child point to mental health and child populations respectively. This reflects the expanded application of digital health interventions in special populations and mental health services. Fourth, the appearance of cluster #3 hiv indicates that digital health interventions also have important applications in HIV prevention and treatment, such as using mobile health apps to improve antiretroviral adherence and promote preventive services among high-risk groups. Cluster #8 electronic health records reflects the supporting role of data infrastructure in digital health. Cluster #1 middle aged, as a demographic cluster, suggests that research in this field focuses on intervention effects in specific age groups. Overall, Q = 0.5525 (greater than 0.3) indicates a significant cluster structure. S = 0.7868 (greater than 0.7) indicates high internal consistency of clusters, meaning that keywords within each cluster are closely related (57), while clusters are clearly distinguished from each other. This knowledge map clearly delineates the research frontiers of digital health interventions across different diseases, populations, and technology dimensions.

### 3.8 Bibliometric laws analysis

To complement the descriptive bibliometric analysis, we applied three classical bibliometric laws—Lotka's Law, Bradford's Law, and Zipf's Law—to further characterize the field's knowledge structure (58).

#### 3.8.1 Lotka's Law (author productivity)

Lotka's Law describes the inverse square relationship between the frequency of scientific output and the number of contributing authors in a given field. Applying Lotka's Law to our dataset of 24,881 authors, we identified the core productive authors and examined whether the observed author productivity distribution conforms to the theoretical Lotka distribution. The results showed that a small number of authors (approximately 6% of all authors) contributed more than 50% of the total publications, indicating a high concentration of research productivity. This pattern is consistent with findings in other health-related bibliometric studies and reflects the existence of “knowledge elites” who shape the research agenda in this field (59).

#### 3.8.2 Bradford's Law (journal dispersion)

Bradford's Law predicts that if journals are grouped into zones of roughly equal numbers of articles, the number of journals in each zone follows a geometric progression (60). We divided the 1,460 journals into three zones, each containing approximately one-third of the total 4,677 articles. Zone 1 (the core zone) consisted of 12 journals publishing 1,559 articles (33.3% of total). The JMIR series journals—Journal of Medical Internet Research, JMIR Formative Research, JMIR mHealth and uHealth, and JMIR Research Protocols—all fell into Zone 1, confirming their role as the core dissemination platforms in this field. Zone 2 contained 68 journals publishing 1,559 articles, and Zone 3 contained the remaining 1,380 journals publishing 1,559 articles. The Bradford multiplier *k* was calculated as approximately 5.7, indicating a moderate level of paper concentration compared to other health research fields. Zipf's Law (Keyword Frequency). Zipf's Law states that the frequency of a word is inversely proportional to its rank. We applied Zipf's Law to both author keywords (DE, *n* = 9,302) and Keywords Plus (ID, *n* = 4,971). The results showed that author keywords deviated significantly from the ideal Zipf distribution, with a long tail of low-frequency terms (only 341 keywords appeared more than five times). This confirms the conceptual fragmentation noted in Section 3.1. In contrast, Keywords Plus exhibited a distribution closer to the Zipf prediction, reflecting the greater standardization of indexer-assigned terms. This discrepancy underscores the urgent need for a unified terminology system in digital health equity research (61).

Together, these three bibliometric laws provide quantitative evidence for the structural characteristics observed in our descriptive analysis: high author productivity concentration, journal core-periphery stratification, and keyword term dispersion (62).

### 3.9 Funding pattern analysis

To understand the structural determinants of research production in this field, we analyzed funding patterns.

#### 3.9.1 Funded vs. non-funded research output

Among the 4,677 documents, 1,842 (39.4%) acknowledged funding from one or more sources. Funded research exhibited significantly higher citation impact (mean citations = 18.7 vs. 12.3 for non-funded, *p* < 0.001) and was more concentrated in Q1 journals (62.3% of funded articles vs. 41.7% of non-funded articles).

#### 3.9.2 Funding and national economic indicators

Publication frequency showed a strong positive correlation with national GDP (r = 0.87, *p* < 0.001) and R&D expenditure as a percentage of GDP (r = 0.79, *p* < 0.001). The United States, with the highest GDP and R&D investment, accounted for 54% of the top ten countries’ total frequency. China, despite its rapid growth in publication volume, has a lower funded-research ratio (32.1%) compared to the US (48.6%) and UK (44.2%), suggesting that funding availability may constrain the scale and impact of research output.

#### 3.9.3 Thematic differences by funding Status

Funded research disproportionately focused on telemedicine (32.4% of funded vs. 21.7% of non-funded), AI applications (18.6% vs. 9.3%), and implementation science (15.2% vs. 7.8%). Non-funded research more frequently addressed basic descriptive studies, feasibility pilots, and local context analyses.

#### 3.9.4 Global health Index correlations

Countries with higher Global Health Index (GHI) scores tended to produce more funded research and more internationally collaborative research. However, several LMICs with relatively low GHI scores demonstrated high non-funded research output, suggesting that researchers in these settings rely on alternative support mechanisms or conduct research with fewer resources (63).

## 4 Discussion

### 4.1 The paradox of exponential growth: academic prosperity and knowledge disorder

Between 2015 and 2025, the annual number of publications in this field jumped from 75 to 1,317, with a cumulative total of 4,677. The exponential fit had an R^2^ of 0.9955 (64). On the surface, this is strong evidence of academic prosperity. However, upon deeper examination, exponential growth reveals an internal crisis of knowledge production. Price's law describes an “explosion period of scientific discovery,” which is essentially a critical point where a discipline shifts from “problem-driven” to “literature-driven” research. When the growth rate of literature exceeds the information processing capacity of individual researchers, knowledge production begins to detach from responding to real-world problems and instead follows the logic of literature's own reproduction.

Specifically, among the 4,677 documents, how many truly address the core question: “Can digital health interventions reduce health equity gaps?” The large gap between the dispersion of author keywords (9,302) and the standardization of Keywords Plus (4,971) suggests that many studies are conceptually self-referential and fragmented (65), lacking a unified problem framework. More worryingly, the average annual growth rate of 33.18% means that the half-life of literature is very short. A large number of studies are submerged by new literature before they can be fully digested, creating a “knowledge forgetting” effect. Early theoretical insights and critiques may be repeatedly “rediscovered” without citation (66). For example, classic discussions of the digital divide [e.g., (66)] have seen declining citation rates in recent literature. Instead, many empirical studies “rediscover” similar conclusions without citing them, leading to a lack of cumulative knowledge. This knowledge disorder beneath the appearance of prosperity requires us to re-examine the academic evaluation system. Publication volume is no longer a reliable indicator of academic contribution. Research needs to shift from a “quantity competition” to a problem-focused deep accumulation (67). Furthermore, the field needs to establish a “core literature library” and a “list of key questions” to guide researchers away from redundant work and focus on solving unresolved problems.

Beyond the quantitative patterns, a qualitative reading of the literature reveals that many studies in this field share similar research designs—cross-sectional surveys, small-scale pilot studies, and secondary analyses of existing datasets—without substantially advancing theoretical understanding or methodological innovation. This “quantity over depth” phenomenon suggests that the exponential growth may reflect academic incentives (e.g., publish-or-perish pressure) rather than genuine scientific progress.

### 4.2 The illusion of interdisciplinarity and the reality of knowledge barriers

The journal dual-map overlay shows that the field's knowledge inputs span four major blocks: Earth and Environmental Sciences, Basic Biomedicine, Clinical Health, and Behavioral Social Sciences. This interdisciplinary map is often seen as a sign of disciplinary maturity. But we must ask: is this “interdisciplinarity” a genuine integration of knowledge or just a mechanical juxtaposition of literature from different disciplines? In the keyword clustering, “digital health” and “health equity” did not form a tight co-occurrence relationship. This suggests that the two discourse systems of technology development and equity analysis are still “talking past each other” (67).

A deeper problem is that the logics of knowledge production in different disciplines have structural conflicts. Information science pursues technical optimization and universality, tending to develop “one-size-fits-all” solutions (68). Health equity research emphasizes contextual sensitivity and heterogeneity, requiring interventions to be tailored to specific populations. These two logics can be reconciled in theory, but in practice, the performance metrics of technology developers (such as download counts, user activity) often diverge from the concerns of equity researchers (such as coverage and benefit among vulnerable groups). The essence of this knowledge barrier is the incompatibility of evaluation systems, journal preferences, and career incentives across disciplines. For example, top computer science conferences (such as CHI, CSCW) favor technical innovation and completeness of user studies, but pay insufficient attention to sample representativeness (does it include low-income people? Are there multiple language versions?). Conversely, public health journals require rigorous epidemiological designs but often gloss over technical implementation details. The differences in review culture lead to interdisciplinary studies facing a “lose-lose” dilemma when submitting manuscripts. Therefore, true interdisciplinarity is not about scholars from different disciplines publishing separately and then citing each other. It requires the establishment of shared problem frameworks and evaluation standards (69). One possible path is to create a dedicated “digital health equity” interdisciplinary journal, mandating that each paper report both technical effects and equity impacts, and invite both technology experts and public health experts to review together. Furthermore, doctoral training programs should encourage co-supervision to ensure that students master both technical and equity analysis perspectives.

A qualitative examination of the content within each disciplinary block reveals that citations across blocks are often superficial—researchers cite methodological papers from other disciplines without engaging with their underlying theoretical assumptions. For example, technology developers frequently cite behavioral science theories (e.g., TAM, COM-B) as justification for intervention design but rarely critically examine whether these theories are appropriate for vulnerable populations. This “citation without integration” pattern suggests that true interdisciplinarity—the co-construction of shared problem frameworks—has not yet been achieved (70).

### 4.3 Concentration of hotspots and systematic silence of blind spots

Keyword clustering identified 10 core themes (71), focusing on telemedicine, chronic disease management (hypertension, diabetes, breast cancer), mental health, and HIV/AIDS. These hotspots reflect the rational allocation of research resources (72) to areas with clear outcome indicators, established clinical pathways, and sufficient funding. However, the collective consequence of rational choices is the formation of systematic blind spots. First, “health equity” itself did not become an independent high-frequency cluster. We must also consider an alternative interpretation: the absence of ‘health equity’ as an independent cluster does not necessarily mean that equity concerns are absent from the literature. It may instead reflect that equity functions as a cross-cutting framework or analytical lens applied to disease-specific or technology-specific clusters (e.g., ‘Does telemedicine reduce disparities in diabetes care?’), rather than a standalone research topic. Our keyword co-occurrence analysis cannot distinguish between these possibilities. This is an inherent limitation of co-occurrence-based clustering: it captures frequency and co-occurrence patterns, not the depth or centrality of a concept's role in research design.

Furthermore, the point about linguistic and regional variation in how equity is framed is important. As noted in the geographic distribution analysis, research from non-English-speaking and non-Western settings may use terms such as ’social inequality,’ ‘poverty,’ ‘racial discrimination,’ ‘oppression,’ or specific inequity names (e.g., ‘racism,’ ‘machismo’) rather than the standardized ‘health equity’ terminology. The English-language indexation bias of our databases may have systematically underrepresented these alternative framings. This is an important limitation acknowledged in Section 4.7 Instead, it appears as a subordinate tag within the “digital health” cluster. This suggests that equity issues have not yet achieved the same academic status as technological innovation (73). Second, the clusters lack keywords directly pointing to equity dimensions, such as “low-income population,” “rural population,” “low education,” and “ethnic minority.” This implies that research focuses more on “average effects of digital health interventions in the general population” than on “heterogeneous effects in vulnerable groups” (74, 75).

This average-oriented approach has fundamental methodological flaws. If a digital health intervention has strong effects in advantaged groups but weak or even negative effects in disadvantaged groups, the average effect might appear “moderately significant,” masking the possibility that the intervention worsens inequality (76). More concerning, the geographic distribution data reveal a center-periphery structure of knowledge production. The United States accounts for more than 54% of the top ten countries’ total frequency (77). India has only one-third of China's output. African countries are completely absent (78). This means that knowledge about “digital health equity” is mainly produced in high-income countries. The “equity issues” defined by these countries (such as differences in insurance coverage, urban-rural broadband gaps) have almost no overlap with the realities of low- and middle-income countries (such as lack of basic electricity, high illiteracy rates, multilingual environments) (79). This geographic concentration of knowledge production may lead research agendas to be dominated by high-income countries, while local contexts and priority issues of low- and middle-income countries are systematically undervalued in mainstream literature. Some scholars call this the academic center-periphery structure, which merits attention in this field. To break this pattern, we need to establish “reverse innovation” mechanisms. We should encourage researchers in low- and middle-income countries to lead the development of digital health interventions (80)and then export successful experiences back to high-income countries. For example, SMS-based health reminder systems successfully used in Africa may be more applicable to low-income communities in high-income countries than expensive smartphone apps (81).

A qualitative content analysis of the papers within the #0 telemedicine and #5 digital health clusters reveals that most studies frame “equity” as a secondary consideration—mentioned in the discussion section as a limitation or future direction, rather than being integrated into the research design, data collection, or primary analysis. Few studies explicitly report intervention effects by subgroups defined by income, education, race/ethnicity, or geographic location. This “equity as an afterthought” pattern reflects a deeper epistemological bias: the field treats equity as a moderating variable to be checked post-hoc, rather than a core analytical lens from the outset (82, 93).

### 4.4 Centralization of collaboration networks and reproduction of knowledge power

The high concentration of betweenness centrality and PageRank in the author collaboration network reveals a structural pattern in knowledge organization. A few highly influential scholars (such as Michie, Yardley, Harris, Kroenke) occupy both high betweenness centrality and high PageRank (83). This means they are structurally positioned as bridges connecting different sub-fields. From the perspective of science and technology studies, this structural position could be interpreted as conferring potential influence over knowledge flow—what some scholars term ‘knowledge gatekeeping.’ However, it is important to emphasize that this is a theoretical inference from the bibliometric data, not a direct measure of gatekeeping behavior. Bibliometric centrality does not prove that these scholars consciously exclude alternative frameworks; it only shows that any research crossing sub-field boundaries is likely to pass through their citation or collaboration networks. This structural pattern facilitates knowledge integration but also carries the risk of potential knowledge power concentration, wherein the research agendas and theoretical preferences of core scholars may be systematically amplified through peer review, citations, and collaboration networks.

This centralization has special implications for health equity research. If knowledge production is dominated by core scholars from high income countries (15), then the power to define “what is an equity issue,” “how to measure equity,” and “which solutions are effective” rests with these scholars. Local knowledge, alternative practices, and critical voices from low and middle income countries may be marginalized (10). More subtly, core scholars’ institutions typically hold more journal editorial seats, conference organizing power, and grant review authority (22). This institutional power further reinforces the reproduction of the existing structure. Breaking this structure requires institutional interventions. Funding agencies should require that international collaboration projects include scholars from low and middle income countries as co principal investigators. Journals should adopt a “geographic diversity in review” principle, ensuring that reviewers from different regions participate in the review process. Additionally, we should encourage the establishment of “decentralized” academic communication platforms (such as preprint servers, open peer review) to reduce the control of power nodes in the traditional publishing system (23).

Drawing on theories of knowledge-power relations, the high betweenness centrality and PageRank of a few core scholars (Michie, Yardley, Harris, Kroenke) indicates that these individuals function as “knowledge gatekeepers”—they not only produce influential research but also control the flow of ideas across sub-fields. Any research that crosses theoretical, methodological, or applied boundaries can hardly bypass these nodes. This structure facilitates knowledge integration but also risks reproducing existing power asymmetries. Scholars from non-English-speaking countries and low- and middle-income countries, despite their publication volumes, rarely occupy such gatekeeper positions. A qualitative review of the editorial boards of the top 10 journals in this field confirms this pattern—the vast majority of editors are affiliated with institutions in high-income, English-speaking countries (82).

### 4.5 The gap between knowledge production and policy translation

Perhaps the most central finding of this study is not any single result above, but a fundamental problem that these results collectively point to: the gap between academic prosperity and policy silence. In the introduction, we raised the issue of an “evidence-practice gap.” Research outputs are increasingly abundant, but the translation channels from research findings to regulatory guidelines, campus interventions, or family advice have never been opened (25). Our bibliometric analysis provides evidence for this diagnosis. First, the concentration of research hotspots and systematic silence of blind spots mean that the evidence policymakers need—"evidence on effects in vulnerable groups"—is precisely missing (24). Second, the illusion of interdisciplinarity and reality of knowledge barriers mean that the dialogue mechanism between technology developers and public health practitioners has not been established (25). Third, the centralization of collaboration networks and reproduction of knowledge power mean that practical wisdom from peripheral regions (such as low- and middle-income countries, rural communities) struggles to enter mainstream knowledge systems (40). Fourth, the severe imbalance in geographic distribution means that most evidence is produced in high-income countries.

Policymakers in low- and middle-income countries face contexts (such as low digital literacy, unstable internet connections, multilingual barriers) for which there is almost no local evidence. This gap is not accidental. It results from the structural contradiction between the incentives of academic production (pursuing publication volume, citation counts, and high impact factor journals) and the needs of policy (timeliness, contextual sensitivity, operability) (59). A deeper reason is that the academic evaluation system has almost no link to policy impact. A scholar's promotion depends on the number of published papers and journal impact factors, not on whether their research is cited or implemented by policy (59–63, 70, 82, 84, 85). Therefore, bridging the gap cannot rely solely on calls to “strengthen translation.” It requires redesigning the academic evaluation system. Funding agencies should include “policy impact” as an indicator in project evaluation [e.g., requiring project proposals to clearly list the policy clauses or guidelines they aim to influence (59–61)]. Journals should encourage the publication of “negative results” and “implementation study reports,” rather than only publishing “statistically significant effects.” More importantly, we need to establish “knowledge co-production” mechanisms, involving policymakers, community representatives, and frontline practitioners from the research design stage. This ensures that research questions align with real-world needs, research methods fit the implementation context, and research outputs are delivered in actionable formats (such as decision memos, implementation toolkits). The UK National Institute for Health Research's (NIHR) “Research Design Service” model is worth emulating. Researchers must provide an “evidence use plan” detailing which policy agencies, through which channels, and in what format the research results will be applied (62).

Following the Synthetic Knowledge Synthesis (SKS) approach, we triangulated our quantitative bibliometric findings with a qualitative assessment of policy relevance. Following the Synthetic Knowledge Synthesis (SKS) approach, we triangulated our quantitative bibliometric findings with a qualitative content assessment of policy relevance. We reviewed the abstracts and full-text discussion sections of the 50 most-cited papers in our dataset to identify whether they included explicit policy recommendations or implementation strategies. We used the following criteria: (a) explicit reference to a specific policy document, guideline, or regulatory framework; (b) actionable recommendations for healthcare providers, health systems, or policymakers; (c) discussion of implementation barriers or facilitators; or (d) specific proposals for intervention scale-up or adaptation. Only 12 of these 50 papers (24%) met at least one criterion. The remaining 38 papers (76%) discussed implications only in general terms or limited their recommendations to future research directions.

This qualitative finding corroborates our quantitative observation of a research-policy gap. However, we emphasize that this is a qualitative interpretation derived from a targeted review of highly cited papers, not a direct bibliometric measurement. The lack of bibliometric indicators for ‘policy impact’ is itself an important methodological limitation of the field. Bibliometric databases do not systematically track policy citations, and existing policy citation indices (e.g., Overton) are not integrated with standard bibliometric tools. Therefore, our conclusion about the research-policy gap is based on triangulation of multiple pieces of evidence: (a) the dominance of efficacy-oriented research in keyword clusters, (b) the absence of implementation-focused keywords, (c) the geographic disconnect between knowledge production (high-income countries) and primary need (LMICs), and (d) our qualitative review of the most-cited papers. “This is a triangulated interpretation, not a directly measured bibliometric finding.” We reviewed the abstracts of the 50 most cited papers in our dataset to identify whether they included explicit policy recommendations or implementation strategies. Only 12 (24%) contained actionable policy guidance. This finding corroborates our quantitative observation of a research-policy gap and suggests that the field's knowledge production is primarily oriented toward academic audiences rather than policymakers or practitioners (85).

### 4.6 Methodological self-reflection: cognitive traps of bibliometric tools

We must first acknowledge a fundamental epistemological limitation of this study. Bibliometric tools reveal large-scale literature structures, but they do not directly demonstrate causal power relations. High betweenness centrality in a collaboration network indicates that a node is structurally positioned to bridge different research groups, but whether this translates into actual ‘gatekeeping'—the power to include or exclude knowledge—remains a theoretical inference that must be supported by additional qualitative evidence. Throughout this manuscript, we have attempted to maintain this distinction: the Results section reports empirical patterns (e.g., frequencies, centrality values, cluster structures), while the Discussion section offers theoretical interpretations of these patterns. However, we acknowledge that in some instances this boundary became blurred, and we have revised the text to ensure greater clarity.

This study used Bibliometrix, SciExplorer, and CiteSpace for bibliometric analysis. These tools have irreplaceable advantages in revealing large-scale literature structures, but we must also acknowledge their inherent cognitive limitations. First, the logical basis of co-occurrence analysis is “high frequency equals importance.” This assumption is questionable from a sociology of science perspective. Some disruptive theoretical innovations may have very low citation rates in their early stages and may be excluded from clusters. Conversely, some hot topics may simply be results of academic fashion or follow-the-crowd research; their high frequency does not represent substantive knowledge contribution (63, 70). In this study, the “digital health” cluster is large, but how many studies truly push the boundaries of theory or practice versus repeatedly testing similar hypotheses? Bibliometric analysis cannot answer this question. It may even reinforce academic fashion over depth through the default assumption of “high frequency equals importance.” We need to acknowledge that this study itself partially relies on that assumption. Therefore, our interpretation of “health equity not becoming a high-frequency cluster” as a lack of research attention should be seen as a relative judgment based on the current bibliometric paradigm, not an absolute truth. Future mixed-methods research should combine qualitative analysis to verify this finding. Second, clustering algorithms (such as LLR, LSI) are highly sensitive to parameters. The g-index (k = 2) and Pathfinder pruning used in this study are routine settings in the field, but different parameter choices could lead to significant differences in cluster number, size, and labels. This means that the “objective structure” presented by the knowledge map is actually a technical product of the researcher's subjective choices (82). Third, the subject labels in the journal dual-map overlay come from journal classification systems (such as WoS subject categories). These classifications have historical contingencies and geographic biases. For example, placing “traditional medicine” under “complementary and alternative medicine” while regarding Western biomedicine as “mainstream medicine” is itself a manifestation of knowledge power. More fundamentally, bibliometrics simplifies knowledge into computable entities (keywords, citations, co-occurrences), while the qualitative dimensions of knowledge production (theoretical innovation, critical reflection, contextual wisdom) cannot be quantified. Therefore, readers should not interpret the map results of this study as an “objective mirror” of the field's structure. Instead, they should understand them as a “constructive representation” mediated by specific technical filters and classification systems. Future mixed-methods research should combine bibliometric analysis with in-depth content analysis, expert interviews, or case studies (84) to compensate for the blind spots of quantitative methods. For example, one could conduct critical discourse analysis on representative papers in high-frequency clusters to reveal their implicit theoretical assumptions and value orientations. Additionally, “counterfactual bibliometrics” methods should be developed, simulating network changes after removing core nodes to identify which knowledge is truly irreplaceable (84).

Fourth, while our application of Lotka's, Bradford's, and Zipf's laws provides quantitative validation of our descriptive findings, these laws themselves have limitations. Lotka's Law assumes a stable author productivity distribution that may not hold in rapidly evolving fields. Bradford's Law is sensitive to the definition of journal zones and the choice of the multiplier *k*. Zipf's Law may not fit datasets with high term dispersion, as observed in our author keyword analysis. Therefore, our law-based analyses should be interpreted as approximate characterizations rather than precise mathematical proofs (86).

Fifth, the SKS-inspired qualitative content analysis we conducted is inherently subjective. Our selection of which papers to analyze qualitatively, which themes to highlight, and which theoretical frameworks to apply involves researcher judgment. To mitigate this bias, we followed the SKS principle of triangulation—cross-validating qualitative interpretations against quantitative patterns. Future research should employ multiple coders and inter-rater reliability tests to strengthen the qualitative component (87).

Sixth, while our qualitative content analysis of the 50 most-cited papers strengthens the SKS-inspired triangulation, this analysis is inherently subjective. Our selection of which papers to analyze qualitatively, which criteria to apply for ‘policy relevance,’ and which theoretical frameworks to use for interpretation involves researcher judgment. To mitigate this bias, we followed the SKS principle of triangulation—cross-validating qualitative interpretations against quantitative patterns. However, we acknowledge that different coders might apply the policy relevance criteria differently. Future research should employ multiple coders and inter-rater reliability tests to strengthen the qualitative component.

### 4.7 Limitations

This study has the following limitations. First, database coverage bias. We only searched Scopus, WoS Core Collection, and PubMed. We did not include Embase, CINAHL, Google Scholar, or non-English literature. This may have missed some regional or language research outputs. Second, document type bias. We only included original research and systematic reviews. We excluded conference papers, dissertations, preprints, and grey literature. This may cause a lag in capturing emerging topics. Third, inherent limitations of bibliometric tools. Parameter choices in CiteSpace (such as g-index, pruning algorithms) have some subjectivity. The clustering results are not the only objective representation. Co-occurrence analysis assumes “high frequency equals importance,” which may marginalize low-cited but disruptive innovative research. Fourth, inability to assess research quality. Bibliometrics does not distinguish study quality. A low-quality paper and a high-quality paper have the same weight in frequency counts. Fifth, limitations in operationalizing health equity. Although our search terms covered dimensions such as the PROGRESS framework, some equity-related studies may have been missed because they did not use standard terms. Future research should combine qualitative content analysis, systematic reviews, and Delphi expert consultations to address these limitations.

Sixth, our search strategy's reliance on English-language terms such as ‘health equity’ may have missed studies that address equity using culturally specific terminology (e.g., ‘poverty,’ ‘racism,’ ‘oppression,’ ‘machismo’) without employing the standardized English keyword. This limitation is particularly relevant given the geographic distribution findings, which revealed severe underrepresentation of non-English-speaking and low- and middle-income country research. Future studies should incorporate multilingual search strategies and region-specific terminology to capture a more complete picture of global digital health equity research.

### 4.8 Implications for research and practice

Based on the key diagnoses from our analysis, we translate the identified structural biases into actionable future directions.

#### 4.8.1 Addressing knowledge disorder

To counter the “exponential growth with knowledge disorder” phenomenon, we recommend: (a) establishing a **Core Ontology for Digital Health Equity Research** to standardize key terminology (e.g., distinguishing “digital health,” “eHealth,” “mHealth,” and “telemedicine”; standardizing equity-related terms); (b) developing reporting guidelines—such as a PRISMA-Digital Health extension—requiring authors to explicitly report intervention effects by relevant subgroups; and (c) creating a living “core literature library” and “key questions list” to guide researchers toward problem-focused deep accumulation rather than quantity-driven publication.

#### 4.8.2 Bridging interdisciplinary barriers

To move beyond superficial interdisciplinarity, we recommend: (a) creating dedicated “digital health equity” interdisciplinary journal sections or special issues mandating reporting of both technical effects and equity impacts; (b) establishing joint review panels comprising both technology experts and public health/equity researchers; and (c) reforming doctoral training to encourage co-supervision across disciplines, ensuring students master both technical and equity-analytic competencies.

#### 4.8.3 Promoting geographic and epistemic diversity

To break the center-periphery structure, we recommend: (a) funding agencies adopting “fairness in collaboration” principles requiring LMIC scholars as co-principal investigators in international projects; (b) journals adopting “geographic diversity in review” policies; (c) supporting “reverse innovation” mechanisms—encouraging LMIC-led development of digital health interventions with subsequent adaptation to high-income settings; and (d) establishing decentralized academic communication platforms (e.g., preprint servers, open peer review) to reduce traditional gatekeeper control.

#### 4.8.4 Bridging the research-policy gap

To close the academic prosperity-policy silence gap, we recommend: (a) funding agencies including “policy impact” as a formal evaluation criterion, requiring project proposals to specify target policy clauses or guidelines; (b) journals actively soliciting and publishing “negative results” and “implementation study reports”; and (c) establishing knowledge co-production mechanisms involving policymakers, community representatives, and frontline practitioners from research design through dissemination, delivering outputs in actionable formats (e.g., decision memos, implementation toolkits) (88, 94, 95).

## 5 Conclusions

Based on 4,677 documents from Scopus, Web of Science Core Collection, and PubMed between 2015 and 2025, this study used Bibliometrix, SciExplorer, and CiteSpace to conduct a systematic knowledge graph analysis of the intersection of digital health interventions, patient engagement/adherence, and health equity. The main conclusions are as follows. First, the literature in this field has grown exponentially. However, behind the exponential growth, our analysis reveals patterns suggestive of what may be termed a risk of ‘knowledge disorder. Author keywords are highly dispersed (9,302 vs. 4,971 for Keywords Plus). The conceptual system is not yet unified. The half-life of literature is very short, with obvious knowledge forgetting effects, and the field lacks cumulativeness.

Second, the geographic distribution shows a “one superpower and multiple strong countries” pattern. The United States leads absolutely with 9,751 publication frequencies. English-speaking countries (US, UK, Australia, Canada) account for 78% of the top ten countries’ total. China is the only non-English-speaking country in the top five, but it mainly participates in domestic collaborations and lacks network leadership. Low- and middle-income countries (e.g., India) publish far less than high-income countries. African countries are almost absent. This “center-periphery” structure of knowledge production contradicts the goals of health equity.

Third, the journal distribution is centered on the JMIR series (four journals accounting for 58.8% of the top ten journals’ publication volume), forming a full-chain publishing ecosystem from feasibility studies, protocol registration, to efficacy validation. At the same time, comprehensive public health journals (such as BMC Public Health, Frontiers in Public Health) have seen rapid growth in publication volume, indicating that the field is integrating into mainstream public health academia. However, traditional top-tier medical journals (NEJM, The Lancet, JAMA) are not yet major publication venues, suggesting the field is still in a “periphery-to-center” transition period.

Fourth, keyword clustering identified ten core themes: telemedicine, digital health, hypertension, type 2 diabetes, breast cancer, mental health, HIV, child health, electronic health records, etc. But “health equity” itself did not form an independent high-frequency cluster. Moreover, there is a lack of direct keywords for equity dimensions such as “low-income population,” “rural population,” and “low education level.” Research focuses more on average effects than on heterogeneous effects in vulnerable groups, indicating systematic blind spots.

Fifth, the author collaboration network has a multi-center structure. Methodologists (Braun, Tricco), theory builders (Michie, Davis), and tool developers (Harris, Kroenke) form core nodes. High betweenness centrality and PageRank indicate the existence of “knowledge gatekeepers.” Although Chinese scholars have high publication volumes, they have not entered the core network, reflecting their structurally marginal position in international collaboration.

Sixth, the interdisciplinary map shows that knowledge inputs to this field span four major blocks: clinical health, behavioral social sciences, basic biomedicine, and earth/environmental sciences. However, there remain knowledge barriers between technology development and equity analysis. Interdisciplinary integration is still at the level of mechanical juxtaposition of literature, not genuine integration of problem frameworks.

Based on these conclusions, we propose the following recommendations: (1) Establish a core literature library and a list of key questions to guide research from quantity competition to problem-focused deep accumulation. (2) Create a dedicated “digital health equity” interdisciplinary journal that mandates reporting both technical effects and equity impacts, and implement cross-disciplinary review mechanisms. (3) Encourage “reverse innovation” led by scholars from low- and middle-income countries to break the center-periphery structure of knowledge production. (4) Funding agencies and journals should adopt “fairness in collaboration” principles and “geographic diversity in review” principles. (5) Establish knowledge co-production mechanisms that involve policymakers, community representatives, and practitioners in research design, bridging the gap between academic prosperity and policy translation. This study provides the first macro-level knowledge graph of the structural characteristics and evolution patterns in the intersection of digital health interventions, patient engagement/adherence, and health equity. It offers methodological references for subsequent researchers and evidence-based decision support for public health policymakers, funding agencies, and journal editors. Future research should further combine qualitative content analysis and prospective Delphi studies to deeply explore the knowledge content and policy priorities of core clusters.

The application of Lotka's, Bradford's, and Zipf's laws provided quantitative confirmation of three key structural features: high concentration of author productivity, a core-periphery stratification of journals centered on the JMIR series, and significant dispersion in author keywords reflecting conceptual fragmentation. These law-based analyses, combined with qualitative content interpretation following the Synthetic Knowledge Synthesis approach, offer a more comprehensive understanding of the field's knowledge structure than quantitative bibliometrics alone could provide (88).

### 5.1 Novel contributions of this study

This study makes several original contributions to the literature. First, it provides the first macro-level knowledge graph of the intersection of digital health interventions, patient engagement/adherence, and health equity, based on 4,677 documents over a decade. Second, it identifies three systematic biases—geographic (center-periphery structure), thematic (equity as an afterthought), and power-structural (knowledge gatekeeper phenomenon)—that have not been previously documented in this field. Third, it applies Lotka's, Bradford's, and Zipf's laws to quantitatively validate the field's structural characteristics. Fourth, it introduces the SKS-inspired triangulation approach to bibliometric health research, demonstrating how quantitative patterns and qualitative interpretations can be integrated (89).

### 5.2 Implications for future research and clinical practice

For researchers, our findings suggest a paradigm shift from manual narrative reviews to data-driven knowledge graph analysis, and from quantity-focused publication to problem-focused deep accumulation. For clinicians and practitioners (90), our findings highlight the need to critically evaluate whether digital health interventions are designed and tested with vulnerable populations in mind, and to demand evidence on subgroup effects rather than average effects alone. For policymakers and funders, our findings call for investment in LMIC-led research, geographic diversity in peer review, and knowledge co-production mechanisms that bridge the gap between academic output and actionable policy guidance.

## Data Availability

The original contributions presented in the study are included in the article/Supplementary Material, further inquiries can be directed to the corresponding author.
